# Impact of Porcine Arterivirus, Influenza B, and Their Coinfection on Antiviral Response in the Porcine Lung

**DOI:** 10.3390/pathogens9110934

**Published:** 2020-11-11

**Authors:** Damarius S. Fleming, Laura C. Miller, Yun Tian, Yonghai Li, Wenjun Ma, Yongming Sang

**Affiliations:** 1 Oak Ridge Institute for Science and Education Oakridge, Oak Ridge Associated Universities, Oak Ridge, TN 37830, USA; damarius.fleming@usda.gov; 2 Virus and Prion Research Unit, National Animal Disease Center, USDA, Agricultural Research Service, Ames, IA 50161, USA; 3 Department of Agricultural and Environmental Sciences, Tennessee State University, Nashville, TN 37209, USA; ytian@tnstate.edu; 4 Department of Diagnostic Medicine and Pathobiology, Kansas State University, Manhattan, KS 66506, USA; yli@vet.k-state.edu (Y.L.); wma@vet.k-state.edu (W.M.); 5 Department of Veterinary Pathobiology and Department of Molecular Microbiology & Immunology, University of Missouri, Columbia, MO 65211, USA

**Keywords:** PRRSV, IBV-S, lung, immune response, differential expression

## Abstract

Interferon (IFN) cytokines induce an autonomous antiviral state in cells of the infected site to restrict virus spreading and critically regulate overall antiviral response. The antiviral state leads to host protection through expression of hundreds of IFN-stimulated genes that restrict viral infection through multiple mechanisms, for example, directly in viral genome degradation and indirectly through cellular metabolic inhibition. Young pigs were split into four treatment groups: control, porcine reproductive and respiratory syndrome virus (PRRSV, also known as porcine arterivirus) infected, influenza B virus (IBV) infected, and IBV/PRRSV coinfection. Lung tissue was collected at 3, 5, and 7 days post infection (dpi) for control, PRRSV and IBV/PRRSV coinfection, and at 3 and 5 dpi for IBV. Transcriptomic analysis, using usegalaxy.org tools, was performed against the S.scrofa 11.1 reference genome. Differentially expressed gene (DEG) analysis was carried out using DeSeq2 based on the model treatment + dpi + treatment:dpi + E. Downstream analysis examined the interaction of DEG at each dpi for over-enriched gene ontology (G.O.) terms and pathways. Comparisons of the infected groups vs. the controls yielded a total of (*n* = 1412) DEGs for the PRRSV group and (*n* = 1578) for the IBV/PRRSV group across all timepoints. The IBV group had (*n* = 64) total DEGs across 3 and 5 dpi. Expression data were considered statistically significant based on false discovery rate (FDR) ⫹ 0.1. Venn diagram comparisons of the DEGs across dpi showed that groups shared only 16 DEGs at 3 dpi, no DEGs were shared at 5 dpi, and for 7 dpi, only the PRRSV and IBV/PRRSV groups were compared and shared a total of 43 DEGs. Across the comparisons, differential expression was observed in antiviral genes such as IRF1, MX1, and OAS2. The IBV and IBV/PRRSV groups showed higher expression of antiviral genes at earlier dpi than the PRRSV group. Additionally, downregulated genes from the comparisons clustered around Kyoto Encyclopedia of Genes and Genomes (KEGG) pathways effecting lung development and cellular integrity. Early expression of host IFN and antiviral genes may lead to viral RNA degradation, and assembly and transcription inhibition in the IBV infections. In comparison, expression of antiviral genes in the PRRSV group decreased across time. The decrease may explain why PRRSV infections persist, while IBV clears. Moreover, all infected groups showed prolonged upregulation in neutrophil degranulation pathway activity, possibly exacerbating symptomatic lung lesion pathology seen in these respiratory infections.

## 1. Introduction

The onset of viral respiratory infections in swine production systems can often lead to rapid spread due to a variety factors that include, but are not limited to, housing, stocking density, and shipping. Additionally, pigs are subject to species-specific respiratory illnesses such as porcine reproductive and respiratory syndrome virus (PRRSV, also known as porcine arterivirus) and zoonotic pathogens such as influenza B [1,2,3]. Previous studies have shown that PRRSV is the more common swine infection of the two respiratory diseases and can develop co-infections with influenza B (IBV) [1]. There is also evidence that PRRSV and porcine influenza A, while sharing some overlap, produced differences in immune related gene expression during the first seven days of infection [4]. During infection, the anti-viral response is achieved through the process of interferon (IFN)-mediated antiviral immunity that leads to virus-restriction in the infected cell and activation of the antiviral state in neighboring cells [5,6]. The antiviral state leads to host protection through expression of IFN-stimulated genes (ISGs) that restrict viruses through translation inhibition, viral degradation, and mRNA assembly and transcription inhibition. The effect of IFNs and ISGs drives the induction of the antiviral state in adjacent cells as a major mechanism of the antiviral innate immunity. For immune cells, neutrophils and macrophages, as part of the early reaction to viral lung infections, act as first responders and along with ISGs use inflammation and phagocytosis to clear pathogens through mediating inflammation, phagocytosis and expression of ISGs and other immune effectors [5,6,7].

Both PRRSV and IBV cause natural infections in pigs, and IBV is a representative zoonotic and anthroponotic virus able to infect humans. Pigs with a coinfection by PRRSV and IBV thus provide a model to study the viral interaction and host immune response commonly faced during viral respiratory diseases. As a primary infection, PRRSV opens the gate for the development of co-infections in swine such as IBV. Although the mechanisms differ, the similarities in illness caused by PRRSV and IBV go beyond acute respiratory symptoms but also have the potential to separately lead to lung tissue damage, acute respiratory distress syndrome (ARDS), and pneumonia. However, part of this lung injury is attributable to mechanisms involved in the host immune response itself, especially when the response is chronic [2,8,9,10]. The antiviral state is a necessary part of the host returning to health; however, when inflammation and other immune factors are extended over time autoimmune injury can occur [8]. There is a balance between the immune response feedback that is underlain by changes in gene expression within the lung. When this balance is lost it can cause the immune system to inadvertently damage host lungs. This in vivo study set out to characterize changes in host ISG expression both specific and common to viral livestock respiratory infections. The candidate genes highlighted in this study will help with understanding IFN signaling and thereby the establishment of the antiviral state in relation to porcine tolerance and susceptibility to an acute respiratory syndrome. Additionally, this study was also able to investigate changes in gene expression related to tissue damage in the lung, which has implications in treating these symptoms in livestock and humans.

## 2. Results

### 2.1. Infections (PRRSV, IBV, and IBV/PRRSV) vs. Control for Lung Samples

Comparison of the PRRSV infected samples vs. the control samples resulted in a total of 1018 statistically significant DEGs (False Discovery Rate (FDR) < 0.1) for 3 dpi (566 upregulated/452 downregulated), 235 DEGs for 5 dpi (167 upregulated/68 downregulated), and 159 DEGs for 7 dpi (105 upregulated/54 downregulated). The statistically significant results for the IBV/PRRSV co-infected samples vs. the controls produced 667 DEGs for 3 dpi (362 upregulated/305 downregulated), 707 DEGs for 5 dpi (452 upregulated/255 downregulated), and 204 DEGs for 7 dpi (152 upregulated/52 downregulated). Surprisingly, the number of DEGs at 3 dpi were lower for the co-infected group than the PRRSV group. Even though there was less differential expression, the co-infected group showed increased interaction between the genes expressed (Supplementary tables). The IBV vs. controls comparisons resulted in a total of 49 for 3 dpi (32 upregulated/17 downregulated) and 15 for 5 dpi (7 upregulated/8 downregulated) statistically significant DEGs. Changes in gene expression for the IBV treatment group were less dynamic than what was observed in the other treatments. This was consistent with the clinical course of symptoms exhibited by the virus. In all, the treatment groups showed marked upregulation of multiple ISGs including the direct antiviral effector genes such as 2’-5’-oligoadenylate synthetase like (OASL), MX dynamin like GTPase 1 (MX1), and interferon alpha inducible protein 6 (IFI6), as well as the effect-amplifying transcription factors such as signal transducer and activator of transcription 1 (STAT1), and (Supplementary Tables S1–S8).

#### 2.1.1. PRRSV vs. Controls

Several IFN-regulatory factor (IRF) genes were consistently upregulated across all three PRRSV infection timepoints consisting of IRF1, IRF4, IRF8 and varied interferon effector induced genes. These ISGs and IRFs are highly expressed in lungs [11] and together help drive the host towards the antiviral state [5].

Data for the PRRSV treatment at 3 dpi displayed more upregulated genes, but also higher fold changes in general for the upregulated genes (Supplementary Table S1). There were 54 genes that had an upregulated fold change of two or greater. Indeed, a large number of chemokine genes were being upregulated at 3 dpi. Many of the gene ontology (G.O.) terms and pathways related to the upregulated genes were impacts on immune processes. Most of the downregulated genes pointed to hindrance of pathways related to homeostasis, binding, extracellular matrix, and lung development. There were a few highly upregulated genes that showed up in multiple pathways and G.O. terms related to adaptive host immunity, immune effector expression, and proteoglycan binding such as cathepsin L (CTSL) found to be high expression in pig lung alveolus [12]. Other upregulated genes of immune interest included genes such as T cell immunoreceptor with Ig and ITIM domains (TIGIT) which is a negative regulator of IL-12 and T-cell regulation, but a positive regulator of IL-10 [11,13]. In all, the 3 dpi results showed upregulation of many anti-viral effector genes such as interferon induced protein 44 (IFI44) that can be activated by interferon regulatory factors [11,13]. Some important genes included upregulation of pulmonary genes, pulmonary surfactant-associated protein A (SFTPA1) and pulmonary surfactant-associated protein D (SFTPD) which are involved in the defense and maintenance of proper lung respiration [14,15,16]. There was also observed downregulation of innate immune and structural activity genes such as, nitric oxide synthase 3 (NOS3) which binds arginine and is involved in lung development and cell redox homeostasis [11,13]; ficolin (collagen/fibrinogen domain containing lectin) 2 (FCN2) involved in innate immune response through complement activation [11,13]; secretory leukocyte peptidase inhibitor (SLPI), and angiotensinogen (AGT).

At 5 dpi there was a marked drop in the number of statistically significant DEGs compared to 3 dpi. Expression changes at 5 dpi showed continued upregulation of host IFNs, cathepsins, and antiviral genes such as OASL, IFN stimulated exonuclease gene 20 (ISG20), and major histocompatibility 1 (MHC I) related genes such as proteasome subunit beta 9 (PSMB9), beta-2-microglobulin (B2M). There was also upregulation of anti-inflammatory genes such as suppressor of cytokine signaling 1 (SOCS1). Downregulation of genes at 5 dpi mostly related to the extracellular matrix such as integrin subunit alpha 8 (ITGA8) and small leucine rich proteoglycans like fibromodulin (FMOD) which also functions as a damage associated molecular pattern signaler (DAMP) and has been observed in previous studies of PRRSV [17]. One of the most downregulated genes was atypical chemokine receptor 3 (ACKR3) a viral response gene related to chemotaxis [11,13].

At 7 dpi the host immune system was showing less upregulation of ISG genes, such as OASL and IRF1. The treatment group switched from innate immune responses to expressing other immune genes to drive toward the antiviral state such as CTSL, TIGIT, coronin (CORO1A), and alveolar macrophage-derived chemotactic factor-II (AMCF-II). There was also a shift in more upregulation of complement and MHC related genes that included MHC class II histocompatibility antigen SLA-DQA (ENSSSCG00000001456), major histocompatibility complex, class II, DR alpha (HLA-DRA), T-cell surface glycoprotein CD8 beta chain (CD8B), and T-cell surface glycoprotein CD8 alpha chain (CD8A). Additionally, there was also continued upregulation of genes involved in lysis and opsonization of antigens. The differential expression at 7 dpi also gave the indication of the infection moving from lungs to the lymph nodes observed by upregulation of multiple granzyme and chemokine genes. Downregulation continued to occur in ITGA8, ACKR3, as well as, homeostasis and immune related genes at 7 dpi that included caveolin (CAV1) and Neuron derived neurotrophic factor (NDNF) [11,13] (Supplementary Tables S1–S3).

#### 2.1.2. IBV/PRRSV vs. Controls

The IBV/PRRSV co-infection group also increased additional expression of several transmembrane ISG proteins that are not seen in the PRRSV single infection such as IFN induced proteins and transmembrane proteins. One of the most respiratory infection relevant being IFN induced transmembrane protein 3 (IFITM3), an antiviral effector exerting potent activity to inactivate influenza virus [18].

At 3 dpi, differential expression of the co-infection was less than what was observed with the PRRSV infection showing almost the same number of statistically significant upregulated and downregulated genes. Many of the upregulated genes at this timepoint indicated a push towards activation of the antiviral state. However, there was a reduced number of IFN stimulated genes only encompassing two, ISG12(A) and ISG15, compared to the PRRSV treatment group which also included ISG20 at the same timepoint. Downregulation was observed mostly in genes related to metabolic, receptor signaling, and cellular structure. The 3 dpi timepoint also saw the exclusion of IRF1, but showed upregulation of IRF4, IRF7, and IRF8 responsible for stimulating antiviral effector genes [5,11,13,19,20].

The number of DEGs increased at 5 dpi and included a greater number of upregulated genes than 3 dpi, but less downregulated genes. Of the upregulated genes there were multiple IFN induced protein genes differentially expressed that included IFN induced transmembrane protein 3 (IFITM3) and IFN induced protein with tetratricopeptide repeats 1,2, and 5 (IFIT1, IFIT2, IFIT5). Compared to 3 dpi upregulation increased in some viral response genes such as IFN-α inducible protein 6 (IFI6) which, even though involved in host viral defense, also has negative regulatory effects on apoptosis. Equally present and upregulated were other IFN-induced antiviral genes. The co-infection allowed observation of multiple genes involved in the obstruction of the viral life cycle within the host such as MX1,MX2, OASL, OAS2, heat shock protein family A (Hsp70) member 8 (HSPA8), and radical S-adenosyl methionine domain containing 2 (RSAD2) [11,13]. One of the largest gene groupings at 5 dpi were 31 ribosomal protein genes that were slightly upregulated (<1 LogFC).

Downregulation was most pronounced in surface receptor signaling and matrix/structural genes that included tenascin (TNC). Moreover, there was downregulation of genes with a negative effect on host immune responses such as Kruppel like factor 4 (KLF4), NDNF, calcium voltage-gated channel auxiliary subunit alpha2delta 1 (CACNA2D1) involved in pulmonary functions, and vascular endothelial growth factor A (VEGFA) involved in chemotaxis and monocytic differentiation [11,13].

By 7 dpi the number of DEGs dropped again. The upregulated genes made up the bulk of the results with only 52 of the 204 being downregulated. The most abundant upregulated gene class at 7 dpi were the ribosomal proteins of which there were 22. The co-infected group was still overexpressing the IFN-stimulated antiviral genes MX1, OASL [5,20], and CCL5 observed in the earlier timepoints and with the PRRSV infected group. There was also continued upregulation of MHC genes indicating a continued adaptive immune response present starting at 5 dpi. Additionally, there was still continued upregulation of cathepsin genes. Downregulated genes of interest included macrophage receptor with collagenous structure (MARCO) involved in pattern recognition and binding of unopsinized particles in the alveolar macrophages, where the gene is expressed based on human annotation. Furthermore, changes in MARCO expression have been linked to pneumonia onset after influenza infections [21]. Other downregulated genes of interest to the host immune response at 7 dpi included pro-platelet basic protein (PPBP) involved in neutrophil chemotaxis and inflammatory response signaling; cellular communication network factor 1 (CCN1) is a gene involved in positive regulation of multiple metabolic processes, matrix stability, and was the most downregulated gene at 7 dpi. The gene CCN1 is also heparin dependent and its lower expression could be linked to downregulation observed in the heparan sulfate proteoglycan 2 (HSPG2), a matrix gene with metabolic and inflammatory actions [11,13].

#### 2.1.3. IBV vs. Controls

Although the number of DEGs decreased greatly within the IBV treatment group, expression at 3 dpi displayed evidence of the animals mounting an antiviral response that included innate and adaptive immune responses early. This included the upregulated genes interferon alpha inducible protein 6 (IFI6) involved in innate immune defense against viral infections and negative regulation of apoptosis; putative ISG12(A) protein (ISG12(A)), also known as IFN-α inducible protein 27 in humans; CD300c molecule (CD300c), a monocytic cell receptor; C-X-C motif chemokine ligand 10 (CXCL10) involved in host viral defense; and DnaJ heat shock protein family (Hsp40) member B1 (DNAJB1), which is shown to aid viral ribonucleoprotein transport in influenza A infections in humans [13,22,23], but has not been shown to do so in swine. Additionally, genes related to binding such as galectin 3 binding protein (LGALS3BP) were upregulated, whereas, the gene galectin 3 (LGALS3), key to binding and immune cell taxis like eosinophil and monocyte activity, was downregulated [13,23]. Other downregulated genes of interest consisted of gelsolin (GSN), an actin binding gene involved in negative regulation of viral entry in mouse lung [23,24]; TRAF interacting protein with forkhead associated domain (TIFA) key to pro-inflammatory activation of NF-kappa-B signaling in humans [25]; and two genes, tetraspanin 14 (TSPAN14) and fatty acid binding protein 5 (FABP5) involved in neutrophil degranulation [13]. The most downregulated gene at 3 dpi was C-C motif chemokine ligand 24 (CCL24), highly expressed in the pig lung and an activator of the host innate immune response in humans [11,13].

The results at 5 dpi consisted of fifteen genes and can likely be attributed to mildness of the influenza B virus in pigs [3]. Unfortunately, many of the genes in this grouping are poorly annotated within the pig genome. Genes of interest differentially regulated at this timepoint included the upregulated genes serine peptidase inhibitor, Kazal type 5 (SPINK5) which is poorly annotated in pigs, but thought to be a component of the immune response involved in protection of mucous epithelia, helping to mount inflammatory responses for humans [26]. In pigs, SPINK5 is biased to the lungs, but in humans it appears to be only expressed in the esophagus and skin [11]. Of the eight downregulated genes heat shock protein family B (small) member 1 (HSPB1) stood out due to its contribution to porcine circovirus 2 (PCV2) infections when upregulated in pigs.

### 2.2. Venn Diagram Results of Genes Shared by All Three Treatment Groups

**Figure 1:**
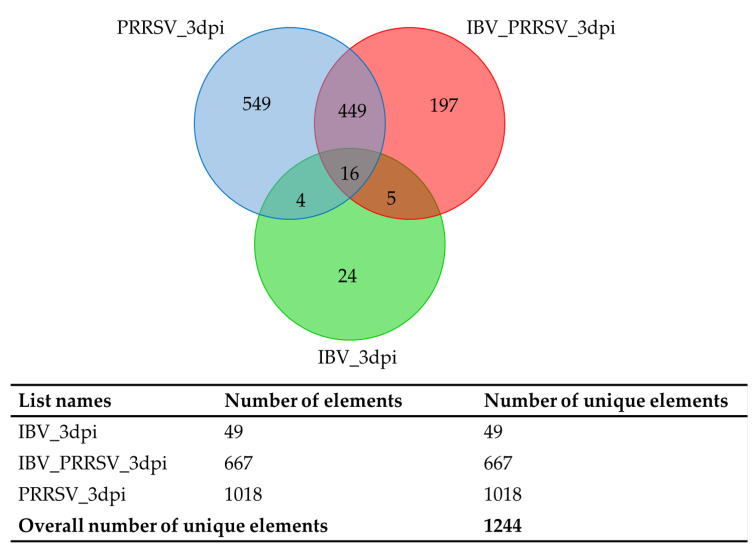
At 3 dpi the Venn diagram shows that the differences in type of respiratory infection are observable with the PRRSV infection showing a greater effect on host immunity early in infection. The shared genes indicate a subset of interferon induced anti-viral genes of general importance to host immune response.

**Figure 2:**
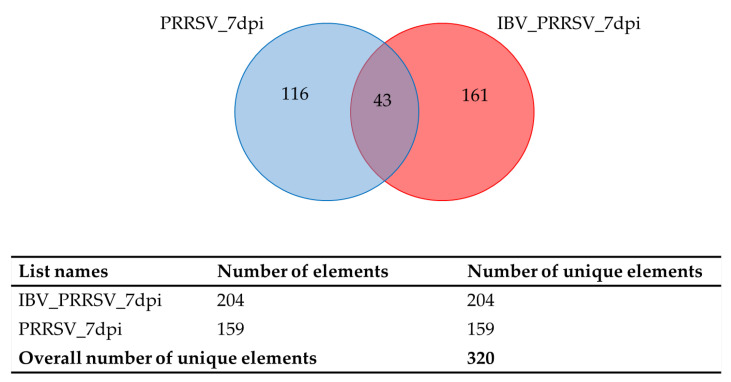
Venn diagram showing number of shared genes between PRRSV and IBV/PRRSV at 7 dpi.

Venn diagram comparisons of the DEGs at each timepoint showed that all three infected groups shared only 16 DEGs at 3 dpi (Table 1 and Figure 1) and no DEGs were shared between the three groups for 5 dpi. However, for 7 dpi, only the PRRSV and IBV/PRRSV groups were compared due to lack of data for IBV at 7 dpi (Table 2 and Figure 2). The two groups shared 43 DEGs. The shared data at 3 dpi highlighted a commonality in the infections to stimulate the antiviral state and set a path towards neutrophil degranulation within the host based on gene individual pathway involvement [11,13]. The 3-dpi data showed more upregulation of the shared genes and some genes had larger expression changes in the single infection group as opposed to the co-infection. One gene, C-X-C motif chemokine ligand 10 (CXCL10), showed an upregulated fold change in the PRRSV single infection that was approximately twice the expression of the coinfected group and three times that of the IBV group. A similar result was also observed for the gene joining chain of multimeric IgA and IgM (JCHAIN) [1,2]. Two genes, interferon alpha inducible protein 6 (IFI6) and C-C motif chemokine ligand 24 (CCL24), had the largest expression change in the IBV group. The shared genes at 3 dpi included a mix of immune effectors, antiviral defense, and structural genes such as MX dynamin like GTPase 1 (MX1), putative ISG12(A), CD300C, IFI6, CXCL10, and JCHAIN [5,11,13,20] (Table 1).

### 2.3. Gene Ontology (GO) and Pathway Analysis of Treatment Groups

To better understand the effect of the changes in gene expression in vivo, the DEGs were examined for statistically significant groupings of processes and pathways.

#### 2.3.1. PRRSV

**Figure 3:**
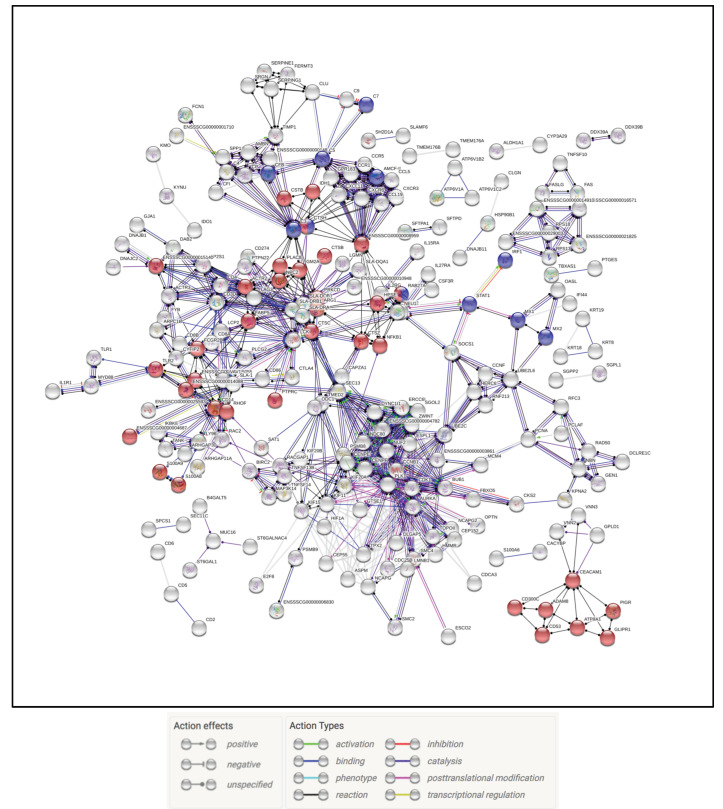
Interaction of upregulated genes in the PRRSV treatment group. Gene nodes in red denote genes part of the neutrophil degranulation process, indicating the process is chronic and possibly linked to lung injury. Gene nodes in blue show immune effector genes.

**Figure 4:**
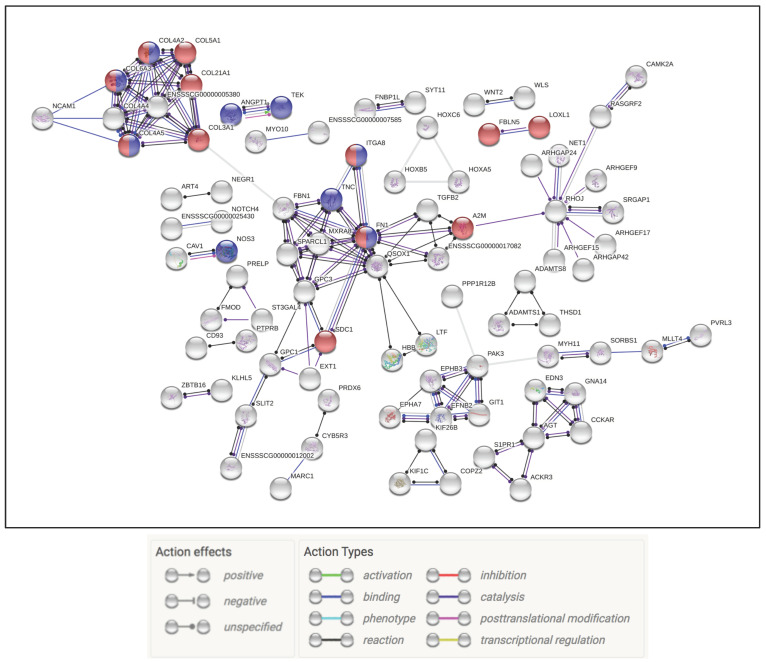
Interaction of downregulated genes across all time points in the PRRSV treatment group. Genes in red and blue represent extracellular matrix (ECM) and degradation genes.

The PRRSV only infection group had the largest gene total at 3 dpi leading to a large number of affected pathways and processes that included upregulated genes falling into categories such as positive regulation of immune effector process(GO: 0002699), negative regulation of viral process (GO: 0048525), neutrophil chemotaxis (GO: 0030593), and ISG15 antiviral mechanism (REAC:R-SSC-1169408). At 5 dpi genes fell into upregulated categories that included positive regulation of immune effector process (GO: 0002699), negative regulation of viral process (GO: 0048525), and also a continuation of neutrophil degranulation (REAC:R-SSC-6798695) observed at 3 dpi. There was also evidence of immune homeostasis dysregulation with up and downregulated genes being statistically significant for immune effector process (GO: 0002252) and reduced respiratory protection with the human Phenotype Ontology term recurrent respiratory infections (HP: 0002205). The categories of interest at 7 dpi continued to show upregulation of both the immune effector process (GO: 0002252), neutrophil chemotaxis (GO: 0030593), and neutrophil degranulation (REAC: R-SSC-6798695) (Supplementary Figures S1–S3). The interaction of the DEGs in the PRRSV treatment group was examined across all time points as either the up or downregulated genes only. The upregulated genes produced an interactome that displayed constant upregulation of genes involved in neutrophil degranulation (REAC: R-SSC-6798695) and immune effector process (GO: 0002699) (Figure 3 and Figure 4).

#### 2.3.2. IBV/PRRSV

**Figure 5:**
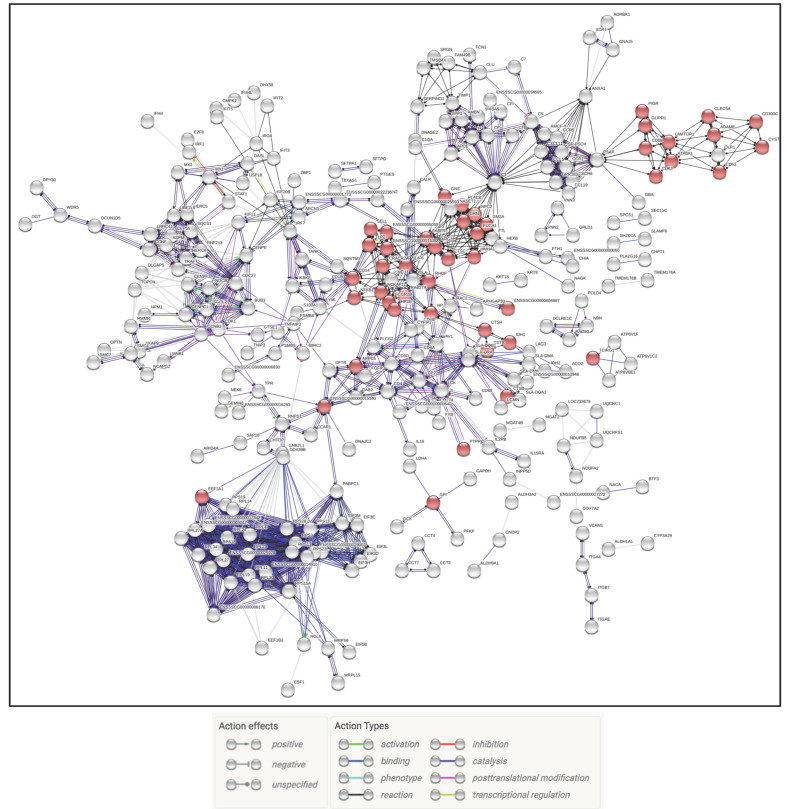
Examination of all of the upregulated IBV/PRRSV genes. Gene nodes in red denote genes part of the neutrophil degranulation process.

**Figure 6:**
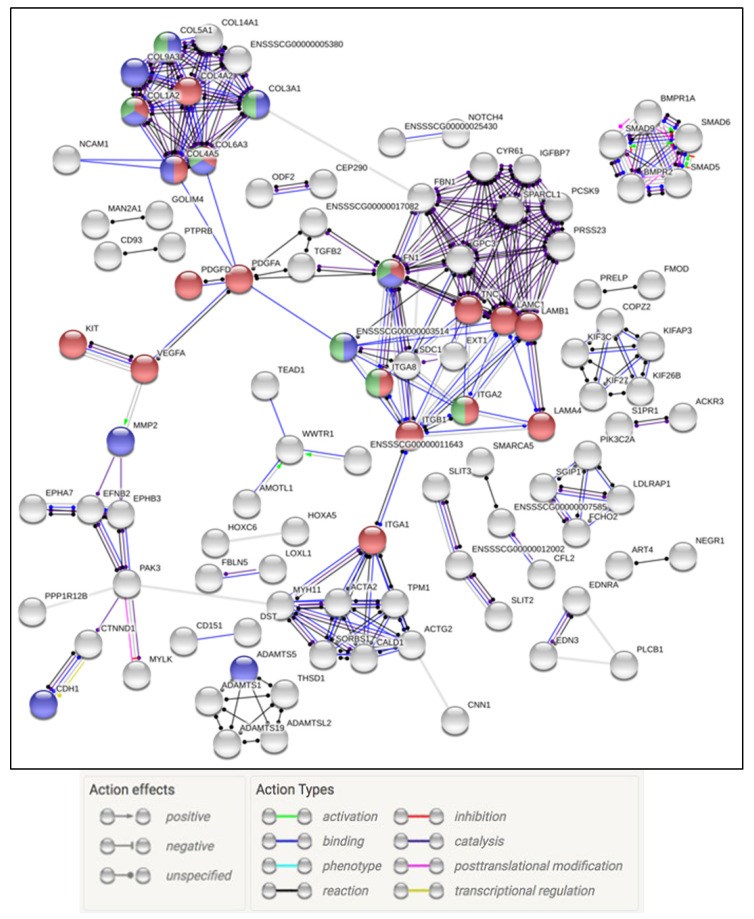
Interaction of downregulated genes across all time points in the IBV/PRRSV treatment group. Red and blue genes represent ECM structure and degradation genes.

At 3 dpi the co-infection group showed a robust multi-faceted immune response that encompassed ramped up innate, complement, and adaptive immune responses. There were 21 upregulated genes related to the defense response to virus (GO: 0051607), 16 for neutrophil migration (GO: 1990266), and 21 for neutrophil degranulation (REAC: R-SSC-6798695). Of the downregulated genes, clustering occurred around pathways, biological functions, and processes that included 67 genes involved in cell surface receptor signaling pathway (GO: 0007166) and 13 in the PI3K–Akt signaling pathway (KEGG: 04151). At 5 dpi there was evidence of IBV replication taking place observed in slight upregulation of genes involved in viral transcription, viral genome replication (GO: 0019079), and viral entry into host cell (GO: 0046718). In addition, at 5 dpi there were 44 genes involved in continued upregulation of neutrophil degranulation (REAC: R-SSC-6798695) and 15 in the Influenza A pathway (KEGG: 05164). Downregulated pathways of interest included G.O. terms related to signaling, cell structure, and stress. This included pathways like extracellular matrix (ECM)–receptor interaction (KEGG: 04512) and cellular response to stress (GO: 0033554). By 7 dpi the co-infection treatment group had a reduction in the number of DEG’s and affected pathways. Still of interest was the continued upregulation of genes involved in neutrophil degranulation (REAC: R-SSC-6798695) (Supplementary Figures S4–S6). The interaction of the DEGs from all time points was separated by up or down regulation, and examined for molecular interactions. The upregulated genes in the coinfection group displayed a large web of interactions with 48 genes falling into the neutrophil degranulation (REAC: R-SSC-6798695) pathway (Figure 5). The downregulated genes formed an interactome that highlighted the PI3K–Akt signaling pathway (KEGG:04151), degradation of the extracellular matrix (REAC: R-SSC-1474228), and ECM proteoglycans (REAC: R-SSC-3000178) (Figure 6).

## 3. Discussion

### 3.1. Swine IFN-Induced Antiviral Response to PRRSV and IBV Driven by Handful of Transcription Factors

Antiviral activity towards PRRSV and the IBV/PRRSV coinfection displayed upregulation of interferon regulatory factors (IRFs) that included *IRF1*, *IRF4*, *IRF8*, expressed in the PRRSV infection and *IRF7* showing up in place of *IRF1* in the coinfection in this study. The genes *IRF1* and *IRF7* have both been shown to have inhibitory effects on the replication of some viruses [3,4]. This may explain the continued overexpression across the timepoints and the addition of the overexpression of *IRF7* in the coinfection. This may also point to chronic IFN stimulation as part of the malaise attributed to cytokine storms during infections [5]. The PRRSV treatment group had increased expression of several IRFs (*IRF1*, *IRF4*, and *IRF8*) but by 7 dpi only one, *IRF1* was still showing upregulation. The IBV/PRRSV group expressed upregulation of one additional interferon regulatory factor, *IRF7*, but had no IRF expression at 7 dpi despite the co-infection. No IRFs were differentially expressed in the IBV treatment group for 3 and 5 dpi, which may suggest that IRF expression occurred early in the course of infection for IBV. However, there was no data for 7 dpi in the IBV group to see if expression occurred later. Host immune response may rely the most on *IRF1* and *IRF7* upregulation as they have shown to be able to induce immune responses to multiple viruses in humans and macaques [6]. It has been shown that PRRS has the ability to block *IRF3* induction of IFN induced viral immune response [7,8], therefore the upregulation of *IRF1* and *IRF7* may likely show host immune response redundancies to compensate for *IRF3* blocking during PRRSV infection. Additionally studies into PRRSV infections have shown changes in *IRF7* expression linked to ISGs, at 5 dpi [9] similar as to what was observed in the current study. In the case of the co-infection group, expression of both *IRF1* and *IRF7* may be the result of the viral sequence and etiology. The large change in the number of DEGs in the PRRSV group from 3 to 5 dpi and the lack of detectable IRF expression in the co-infection could indicate the difference in timing that a host is able to mount an IFN response. In PRRSV the IRF response may be prolonged to account for viral suppression of IFN signaling [10,11]. The gene *IRF8* has also been shown to exhibit suppression of immune response genes and neutrophils, while promoting monocytic cell proliferation [12,13]. This could possibly exist as another mode for PRRSV to stimulate macrophages to aid in host infections or could point to immune balancing of IFN responses prior to 3 dpi. The gene *IRF4* is considered similar in sequence to *IRF8*, however *IRF8* overexpression could be a pathway in reducing neutrophilic related lung injuries.

### 3.2. Analysis Reveals Candidate Immune Effector Genes Involved in Promoting Host Antiviral State

Differential expression of the genes in IFN-mediated pathways across timepoints of the infections is interesting. This was a remarkable observation since the treatment groups did share expression changes in IFN-stimulated antiviral genes across all infected groups at 3 dpi. Although, PRRSV shows the most kinetic changes in IRF expression, the shared antiviral genes at 3 dpi displayed a common group of differentially expressed antiviral ISG effectors. These genes, including *ISG12(A)*, *MX1*, *IFI6*, *JCHAIN*, and *CXCL10*, shared gene overexpression across the treatment groups representing broad acting antiviral genes. The gene *MX1* is an essential innate immune response gene to a wide variety of viral pathogens and key to host responses to influenza. The gene functions to hinder viral replication and has variations within pigs that contribute to PRRSV resistance [1,14]. The ubiquity of *MX1* may prove useful in identifying the importance of lesser studied co-expressed genes for potential immune actions. The chemokine *CXCL10* has also been well studied for its role in viral immune responses, functioning as an inflammatory gene involved in neutrophil and T-cell chemotaxis [1,15]. The effector *IFI6* does not have many proven functions in pigs, but in humans it is involved in IFN induction and signaling as part of the innate response to viral infections. In human viral infections, *IFI6*’s relation to host immunity appears to be disputable as two separate studies, one touting suppression of hepatitis C virus [16] and the other study claiming *IFI6* upregulation permitted hepatitis C viral entry [17], show the gene’s involvement in viral infections. Although the human viral results are ambiguous in action, the pig immune overexpression of *IFI6* is consistent across two structurally different respiratory infections in the current study. In the current study, *IFI6* is most highly expressed by the IBV group and may point to IFN action that involved in early resolution of IBV in pigs [18,19,20]. This overexpression could possibly lead to a better viral response by the innate immune system against IBV in pigs than what has been observed against PRRSV infections. This decrease in the level of expression of antiviral state related genes in the PRRSV group across time, may partially explain why PRRSV infection persists on farms while IBV clears sooner. There were multiple differentially regulated ISG genes with *ISG12(A)* being the most observable across time and treatment groups. It has been shown to hinder PRRSV infection in vitro up to 2 dpi [21]. The current study indicates that it is upregulated as late as 3 dpi in PRRSV treatment group and 7 dpi in the co-infected group. In general, *ISG12(A)* functions as a viral defense gene and may also be involved in chronic interferon process and apoptotic signaling that damage lung tissue.

The gene *JCHAIN* is involved in both innate and adaptive immune responses and can perform respiratory burst actions in the destruction of pathogens [1]. The pattern of expression and action of these genes may increase host oxidative and vascular stress from respiratory pathogens and lead to lung injury.

### 3.3. Host Immune Response Spotlights Complement and Neutrophil Degranulation Pathways in PRRSV and PRRSV/IBV Infections

Monocytic and neutrophilic cell activation during infections work in union as part of the initial immune response. Acute PRRSV infections may commonly become a chronic/persistent infection in commercial adult pigs and it is possible that continued effector gene induction by ISGs leads to increased neutrophil taxis and degranulation causing subsequent endothelial tissue damage [22]. Currently there is limited information as to what extent neutrophils are part of the antiviral defense to respiratory infections in pigs. In PRRSV infections, it is the macrophages that play a critical factor in the ability of the pathogen to subvert host immunity and proliferate PRRSV. It may be possible that in the case of PRRSV and the IBV/PRRSV co-infection that the neutrophils are playing a larger role in the innate immune response to viral infection. Early stages (3–5 dpi) of the PRRSV and coinfection IBV/PRRSV show dysregulation of host calcium transport and utilization based on expression changes in multiple metabolic genes such as triggering receptor expressed on myeloid cells 2 (*TREM2*), which is a neutrophil receptor involved in chemotaxis and the proper activation of monocytic cells including macrophages. Upregulation of *TREM2* early in infection could be a double-edged sword for the host as PRRSV infects macrophages [11]. Many of the shared genes from the 7 dpi Venn diagram show functions related to immune repair of tissue, possibly leading to fibrosis and causing the host immune system to inadvertently damage host lungs. Genes such as arginase 1 (*ARG1*), were shown to be dysregulated in the alveolar macrophages during PRRSV infections, and integrin subunit alpha 8 (*ITGA8*) has been implicated in lung fibrosis [23,24]. There is evidence indicating that the antiviral response to PRRSV and the IBV/PRRSV co-infection uses a combination of ISGs and IRFs such as *IRF1*, *IRF4*, and *IRF7* to drive the host response. The combination of these gene groups may then lead to an overproduction of neutrophilic responses that, upon activation of granulation, leads to lung injury. In the co-infected group, the upregulation of a cluster of chemokine genes involved in the migration of neutrophils may be part of the immune response that leads to extended neutrophil degranulation and lung lesions. Although upregulation of *IRF8* is thought to switch off host production of neutrophils in the transition to dendritic cell production [12,25], the pathway and G.O. analyses show the process of neutrophil degranulation is still active across timepoints in both the PRRSV and IBV/PRRSV groups. There was also an observed upregulation of granzyme genes at 3–7 dpi (*GZMH*, *GZMA*) and of complement genes that under classical pathway activation release granules from opsonization of the pathogens and can exacerbate lung tissue damage. The co-infection group also displayed differential expression of genes that provided insight into respiratory illness and autoimmune lung damage. Neutrophil degranulation has been previously implicated in contributing to lung damage from the host immune response to a respiratory infection [22,26,27]. For PRRSV and its co-infections this damage may be linked to the overexpression of ISGs that work to establish an antiviral state in infected and adjacent cells. In this study, multiple immune effector genes involved in the establishment of this state were also observed to play parts in neutrophil chemotaxis, migration, and degranulation. The process of degranulation when prolonged over time points can damage endothelial tissue through inflammation and the proteolytic action of the granules [22,26]. All of the treatment groups showed a skewing towards upregulation, which would allow for the utilization of antiviral defense responses in the host, but may contribute to the respiratory injury experienced in some infections. It is possible that the lung lesions that can develop from these respiratory infections will be exacerbated by upregulation of genes involved in neutrophil degranulation. The process of neutrophil degranulation will release granules with the ability to destroy the pathogen, but also can destroy host collagen and fibronectin. The effect on lowly pathogenic infections such as IBV appears to be early clearance and resolution with the possible generation of microlesions causing less damage [18]. For PRRSV, however prolonged degranulation might increase the formation of macro-lesions and respiratory failure during high pathogenic infections. The preponderance of neutrophil related gene expression could be related to normal innate immunity sentinel surveillance or related to symptomatic lung lesion pathology related to respiratory infections. For all three treatment groups the neutrophil degranulation pathway seems to be active early, possibly leading to tissue damage in the lungs due to neutrophil driven inflammatory responses to chemotactic DEGs. The PRRSV and IBV/PRRSV co-infection manifest the DEGs of primary neutrophil granules with protease functions called cathepsins (Supplementary Tables S1–S6). In total there were six consistently upregulated cathepsins *CTSL*, *CTSZ*, *CTSB*, *CTSH*, *CTSD*, and *CTSC*. There was no differential expression of *CTSG*, a recognized neutrophil protease [28,29]. The PRRSV infected and co-infected samples show upregulation of multiple cathepsin genes at all three timepoints. The majority of these cathepsins are acidic cysteine proteases that have the effect of remodeling or degradation of the extracellular matrix which could cause breakdown of lung tissue if prolonged. One of the highest expressed cathepsins in the study, *CTSL*, is best annotated in human and is a protease with structural and immune functions [1]. The upregulation of *CTSL* and *CTSB* may be driven by the heightened macrophage response within the lungs and this may lead to proteolytic breakdown of the tissue matrix and lead to lung injury or lesions in pigs. Cathepsins and neutrophils both contribute to the presence of secretory granules during infection, however *CTSL* can also break down lung elastin that can also lead to tissue damage [28,29]. These genes form interaction networks with multiple immune effector genes including the complement response through interactions with complement 3 (*C3*) involved in pathogen opsonization. Complement activation may therefore play a central role in the host response to PRRSV infections and co-infections. The DEG related to complementation would increase the phagocytosis and may prolong neutrophil degranulation. Part of the injury to host lung tissue during certain respiratory infections may be linked to this immune response. It is difficult to say what role the expression of *CTSL* played in the immune response because although there is evidence of the gene promoting protection against influenza A, it has also been shown to assist the SARS-COV-1 virus in cell entry for replication [30]. Due to PRRSV and SARS-COV-1 both being nidoviruses it likely indicates that the peptidase activity of *CTSL* may be broadly involved for nidoviral respiratory infections [30]. In the PRRSV and IBV/PRRSV co-infection, upregulation of genes and pathways that in the process of host defense, when prolonged at a cellular level for seven days, may add to the host tissue damage and limit recovery. The issue may be related to a combination of host inflammatory response, complement opsonization, reactive oxygen species (ROS), and neutrophil degranulation all contributing to lung damage. Neutrophil chemotaxis by the ISGs leads to the release of secretory granules that clears pathogens, but also can lead to destruction of collagen and other extracellular matrix proteins. This process within the host lung endothelium when coupled with other ISG and cytokine functions can increase the chance of tissue damage. A key gene implicated to contribute to neutrophilic lung damage was *CXCL10* which was overexpressed across multiple timepoints for all treatments. The DEG observed also indicate toxic neutrophils going through granulosis, a possible measure of infection severity.

## 4. Materials and Methods

### 4.1. Animals

Samples were provided by Dr. Wenjun Ma, Kansas State University. Pigs were prepared using a similar method as is described in Ran et al. 2015 [1]. Briefly, 36 pigs (3 to 4-week-old) seronegative for influenza A, influenza B, and PRRSV were split into 4 groups: one for blank control group (group 1) and three infected groups (group 2–4). Each pig from groups 2 and 3 was intranasally infected with the Type 2 PRRSV NPB strain at a tissue culture infectious dose (TCID) of 2 × 10^5^ TCID_50_/mL. Pigs from group #1 and #4 were mock-inoculated with virus-free minimum essential medium (MEM) through the same route. At 14 dpi post infection with PRRSV, each pig from group #3 and #4 was inoculated intratracheally (1 mL) and intranasally (1 mL) with B/Brisbane/60/2008 virus at 10^6^ TCID_50_/mL: group “IBV/PRRSV” and group “IBV”. The remaining Control and PRRSV-infected pigs were inoculated with same volume of virus-free MEM through the same routes: group “Control” and group “PRRSV”. Three pigs from each of the four groups were necropsied at 3 dpi, 5 dpi, and 7 dpi post IBV inoculation. Clinical signs and body temperatures were recorded daily. During necropsy the lungs were removed from pigs. Clinical and pathology results from the treatments were collected and will be presented in a subsequent manuscript. The animal study was reviewed and approved by the Institutional Animal Care and Use Committee at Kansas State University (IACUC#3821, approved on November 19, 2016) and was performed in Biosafety Level 2+ animal facilities under guidance from the Comparative Medicine Group at Kansas State University.

### 4.2. Sample Preparation and Sequencing Analysis

The data was collected from young pigs split into four treatment groups (control, PRRSV, IBV, and IBV/PRRSV) and three time points (3, 5, and 7 dpi). Each time point was represented by three replicates for each treatment group except for the 7 dpi IBV infected pigs. This time point was omitted due to lack of replicates. The study examined the expression of lung tissue samples.

Total RNA was extracted from the samples in-house and RNA quality was measured using the Agilent bioanalyzer to check RIN numbers to assess quality. The library preparation and sequence generation were prepared at the Iowa State University Genomics center using the 3′ Quantseq fwd kit and the Illumina Hiseq 4000. The sequencing produced 100bp single-end reads.

Analysis of the sequencing reads was conducted using tools present at usegalaxy.org [31]. Quality control for the reads was performed using FastQC (http://www.bioinformatics.babraham.ac.uk/projects/fastqc/) [32] to examine the raw read data and counts. Next TrimGalore version 0.6.3 (https://www.bioinformatics.babraham.ac.uk/projects/trim_galore/) [33] was used to remove the adapters and reads with a phred score below 20 prior to aligning them to the Sscrofa 11.1 reference genome [2] using Hisat2 version 2.1.0+galaxy5 (https://github.com/galaxyproject/tools-iuc/blob/master/tools/hisat2/hisat2.xml) [34] set to default parameters for forward strand alignment. Raw counts were generated using FeatureCounts [35] and the Ensembl Sscrofa11.1.98 GTF file [2]. The FeatureCounts tool was set to default parameters. The differential gene expression (DEG) analysis was carried out using DeSeq2 version 2.11.40.6 (http://bioconductor.org/packages/release/bioc/html/DESeq2.html) [36]. The parameters were set to use poscounts for the estimateSizeFactors to account for genes with zero counts and the fit type used was parametric. All other parameters were set to the default. The DEG analysis was based on the model treatment + dpi + treatment:dpi + E. The emphasis was centered on the interaction effect of treatment:dpi and represents the list of statistically significant (FDR < 0.1) genes reported. Gene ids are based on Ensembl id’s (Supplemental Tables S1–S8). Genes listed within the results tables and discussions were converted to gene names using Ensembl Biomart [2] and g:Profiler [37]. Gene annotation was performed through use of the National Center for Biotechnology Information (NCBI) and Uniprot and based on both pig and human [1,15].

### 4.3. Gene Ontology (G.O.), Over-Enrichment, and Pathway Analysis of DEG for Each Treatment by dpi and for Shared Gene Lists from Venn

Further analysis of the interaction of treatment and time for each infection group examined the DEG at each dpi for over-enriched G.O. terms and pathways. This analysis was conducted using the g: Gost function of g: Profiler and STRING DB version 11 (https://biit.cs.ut.ee/gprofile) [37,38]. Use of the g: Gost function allowed for over-enrichment analysis incorporating Molecular Function, Biological Process, Cellular component G.O. terms, reactome, and Kyoto Encyclopedia of Genes and Genomes (KEGG) pathway databases for Sus Scrofa [39,40]. The settings used to interrogate the DEG lists included all known genes as the statistical background for comparison, a Benjamini and Hochberg FDR of < 0.05, and the data sources were left in the default settings. The STRING DB tool was used to examine the interconnectedness of the DEGs. The tool parameters were set to examine genes molecular actions based on query genes only, all active interaction sources available within the tool, and a minimum interaction score of 0.900. Additional analysis of the network interactions was based on an FDR of < 0.1 for statistical significance.

## 5. Conclusions

Taken together these genes may highlight a core set of respiratory infection response genes for diagnostic and treatment innovations in pigs. Across all of the comparisons we could observe from changes in gene expression and the subsequent affected pathways that PRRSV and the IBV/PRRSV coinfection change the ability of the lung to maintain cell-cell junctions, prevent and repair tissue damage, and overall barrier function leading to homeostatic loss. Upregulation of genes that related to IFN persistence and chronic neutrophil degranulation may increase lung lesion and injury in PRRSV infections and acute respiratory distress syndrome (ARDS) related to other respiratory infections. Early expression of host IFN and antiviral genes may lead to the antiviral state associated with effective immune action and metabolic arrest to inhibit IBV infection. In comparison, expression of antiviral genes in the PRRSV group decreased across time. The decrease may explain why PRRSV infections persist, while IBV clears. Moreover, all infected groups showed prolonged upregulation in neutrophil degranulation pathway activity, possibly exacerbating symptomatic lung lesion pathology seen in these complicated respiratory infections.

## Figures and Tables

**Table 1:** The 3-days post infection (dpi) shared gene list across treatment groups.

Gene ID ^1^	Gene Name	log2(FC)-PRRSV	log2(FC)-IBV/PRRSV	log2(FC)-IBV
ENSSSCG00000037508	GSN	−0.94	−1.15	−0.87
ENSSSCG00000034570	IFI6	1.39	1.58	1.92
ENSSSCG00000036383	LGALS3BP	1.39	1.33	1.31
ENSSSCG00000032474	CXCL10	3.28	1.51	1.08
ENSSSCG00000016263	LOC100517129	1.08	1.22	0.75
ENSSSCG00000033909	GIMAP1	0.80	1.12	0.72
ENSSSCG00000035379	JCHAIN	3.34	1.76	1.29
ENSSSCG00000014540	FTH1	1.38	0.98	0.95
ENSSSCG00000036403	FAM180A	−1.55	−1.63	−1.24
ENSSSCG00000035297	ISG12(A)	0.86	1.46	1.46
ENSSSCG00000003682	ANKRD12	1.00	0.95	0.8
ENSSSCG00000016092	SGO2	1.76	1.29	1.08
ENSSSCG00000023296	CENPE	1.98	1.95	1.19
ENSSSCG00000017236	CD300C	1.56	1.19	1.15
ENSSSCG00000035736	CCL24	−1.03	−1.02	−1.26
ENSSSCG00000012077	MX1	0.98	1.72	0.88

^1^ Genes values in red showed higher expression in the single vs. the co-infection.

**Table 2:** The 7-dpi shared gene list across porcine reproductive and respiratory syndrome virus (PRRSV) and influenza B virus (IBV)/PRRSV treatment groups.

GeneID ^1^	Gene Name	log2(FC)-PRRSV	log2(FC)-IBV/PRRSV
ENSSSCG00000036224	ENSSSCG00000036224	2.27	2.23
ENSSSCG00000017705	CCL5	1.77	0.93
ENSSSCG00000008973	NAAA	1.22	1.14
ENSSSCG00000004195	ARG1	1.3	1.64
ENSSSCG00000029414	FCN1	1.4	1.49
ENSSSCG00000035379	JCHAIN	1.49	1.08
ENSSSCG00000032857	S100A12	0.84	0.71
ENSSSCG00000011046	ITGA8	−0.96	−0.76
ENSSSCG00000004336	EPHA7	−1.32	−1.13
ENSSSCG00000001463	PSMB9	0.94	0.84
ENSSSCG00000023374	SRGN	0.73	0.89
ENSSSCG00000016903	GZMA	1.3	1.15
ENSSSCG00000037645	COTL1	0.91	0.75
ENSSSCG00000032383	ENSSSCG00000032383	−0.81	−1.05
ENSSSCG00000013901	IFI30	0.87	1.21
ENSSSCG00000021084	S100A6	0.75	1.02
ENSSSCG00000010554	SCD	0.78	0.63
ENSSSCG00000001770	CTSH	0.74	0.96
ENSSSCG00000002004	PSME2	0.8	0.94
ENSSSCG00000040981	GMFG	0.68	0.81
ENSSSCG00000001453	HLA-DRA	0.8	1.02
ENSSSCG00000002366	NPC2	0.62	0.91
ENSSSCG00000001456	ENSSSCG00000001456	0.84	1.29
ENSSSCG00000015089	JAML	1.06	1.07
ENSSSCG00000006153	FABP5	0.69	0.92
ENSSSCG00000036096	ENSSSCG00000036096	1.03	1.22
ENSSSCG00000009216	SPP1	1.12	1.08
ENSSSCG00000035195	HNMT	0.88	1.03
ENSSSCG00000006800	CD53	0.73	0.8
ENSSSCG00000014540	ENSSSCG00000014540	0.73	0.76
ENSSSCG00000023479	ENSSSCG00000023479	−0.8	−0.72
ENSSSCG00000007435	PLTP	1.06	1.23
ENSSSCG00000025618	TAP1	0.82	0.76
ENSSSCG00000004687	B2M	0.61	0.65
ENSSSCG00000015045	NCAM1	−0.7	−0.88
ENSSSCG00000001396	ENSSSCG00000001396	0.78	0.86
ENSSSCG00000037358	HPS5	1.01	1.08
ENSSSCG00000035820	TXNDC17	0.64	1.01
ENSSSCG00000014051	TSPAN17	0.77	0.9
ENSSSCG00000001502	RPS18	0.49	0.66
ENSSSCG00000036618	ENSSSCG00000036618	0.58	0.71
ENSSSCG00000007585	ACTB	−0.81	−0.88
ENSSSCG00000036224	ENSSSCG00000036224	2.27	2.23
ENSSSCG00000017705	CCL5	1.77	0.93

^1^ Genes values in red showed higher expression in the single vs. the co-infection.

## Supplementary Materials

The following are available online at https://www.mdpi.com/2076-0817/9/11/934/s1, Figures S1–S6: Summary of gene ontology multiquery. Tables S1–S8: Differential expression.
