# Supplementary material for: Impact of Porcine Arterivirus, Influenza B, and Their Coinfection on Antiviral Response in the Porcine Lung
Source: Pathogens. 2020 Nov 11;9(11):934. doi: 10.3390/pathogens9110934 (PMC7697066; doi:10.3390/pathogens9110934)
Supplement: Supplementary file 1 [file pathogens-09-00934-s001.zip › PRRSV_FLUB_SUPPLEMENTARY_FIGURES_9_21_20.pdf]

PRRSV VS. Control 3 DPI: G.O. Multi-query

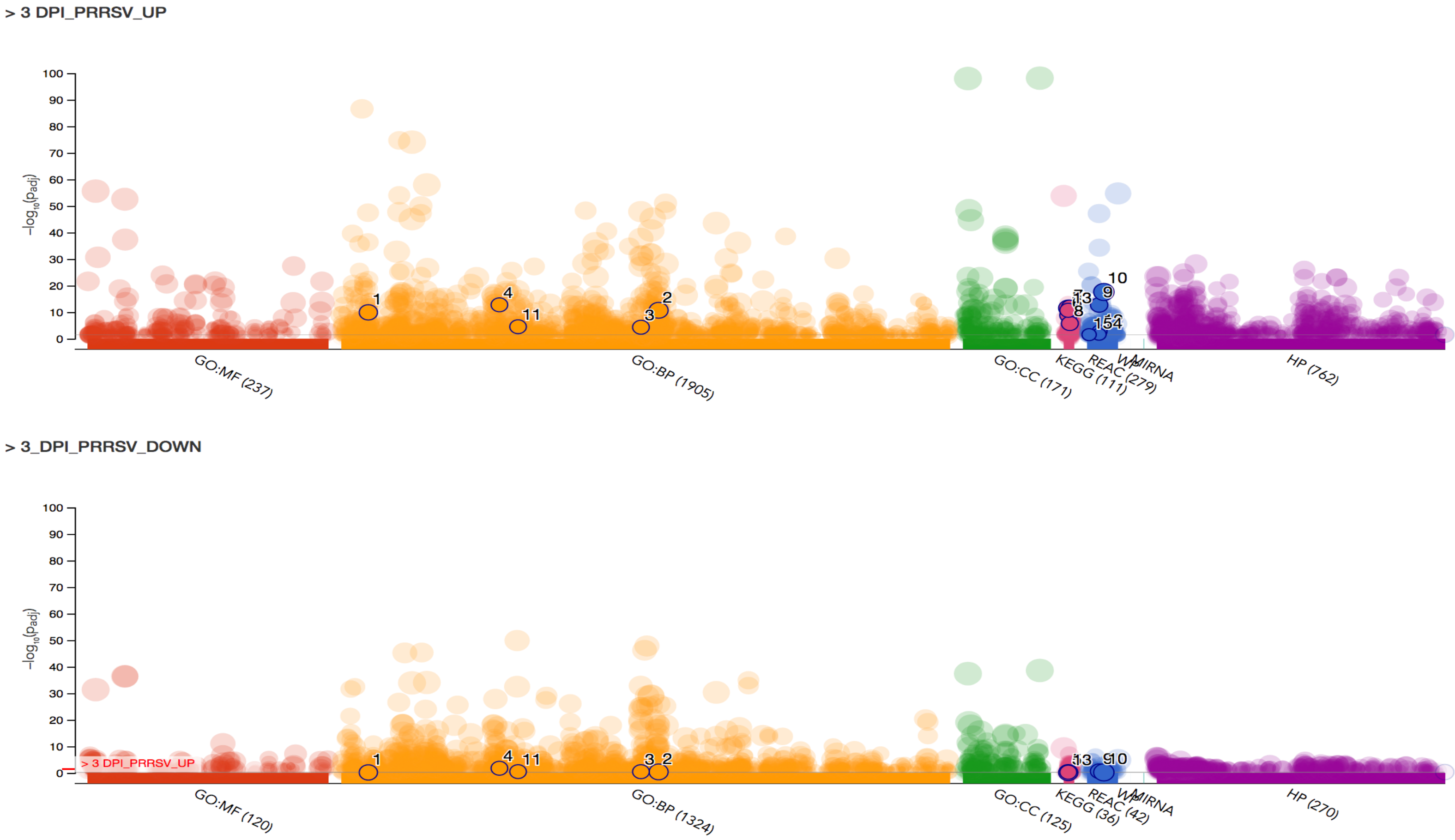

ID	Source	Term ID	Term Name	p_{adj} (3 DPI_PRRSV_UP)	p_{adj} (3_DPI_PRRSV_DOWN)
1	GO:BP	GO:0002699	positive regulation of immune effector process	1.237×10^{-10}	~~6.783×10^{-1}~~
2	GO:BP	GO:0051251	positive regulation of lymphocyte activation	2.088×10^{-11}	~~4.204×10^{-1}~~
3	GO:BP	GO:0048525	negative regulation of viral process	4.983×10^{-5}	~~3.510×10^{-1}~~
4	GO:BP	GO:0030593	neutrophil chemotaxis	1.814×10^{-13}	1.772×10^{-2}
5	KEGG	KEGG:04060	Cytokine-cytokine receptor interaction	3.140×10^{-12}	~~7.687×10^{-1}~~
6	KEGG	KEGG:04064	NF-kappa B signaling pathway	1.460×10^{-9}	
7	KEGG	KEGG:04612	Antigen processing and presentation	9.760×10^{-13}	
8	KEGG	KEGG:04658	Th1 and Th2 cell differentiation	1.884×10^{-6}	
9	REAC	REAC:R-SSC-198933	Immunoregulatory interactions between a Lymphoid and a non-Lymphoid cell	1.953×10^{-13}	~~2.311×10^{-1}~~
10	REAC	REAC:R-SSC-6798695	Neutrophil degranulation	2.626×10^{-18}	~~6.459×10^{-1}~~
11	GO:BP	GO:0032606	type I interferon production	3.225×10^{-5}	~~3.312×10^{-1}~~
12	REAC	REAC:R-SSC-913531	Interferon Signaling	1.589×10^{-3}	
13	KEGG	KEGG:04061	Viral protein interaction with cytokine and cytokine receptor	2.082×10^{-11}	~~5.470×10^{-1}~~
14	REAC	REAC:R-SSC-1169408	ISG15 antiviral mechanism	2.370×10^{-2}	
15	REAC	REAC:R-SSC-1169410	Antiviral mechanism by IFN-stimulated genes	3.094×10^{-2}	

PRRSV VS. Control 5 DPI: G.O. Multi-query

> 5_DPI_PRRSV_UP

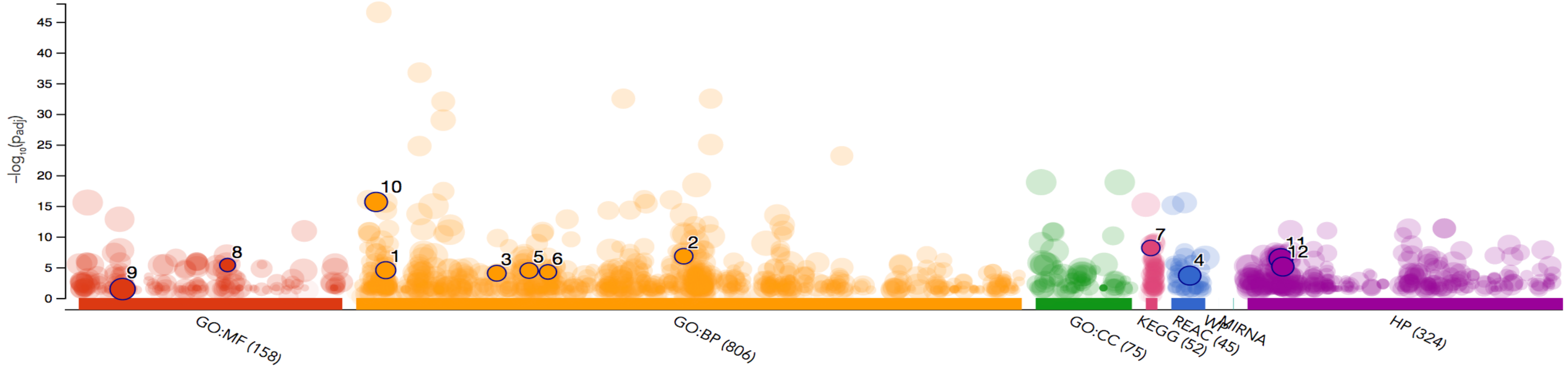

> 5_DPI_PRRSV_DOWN

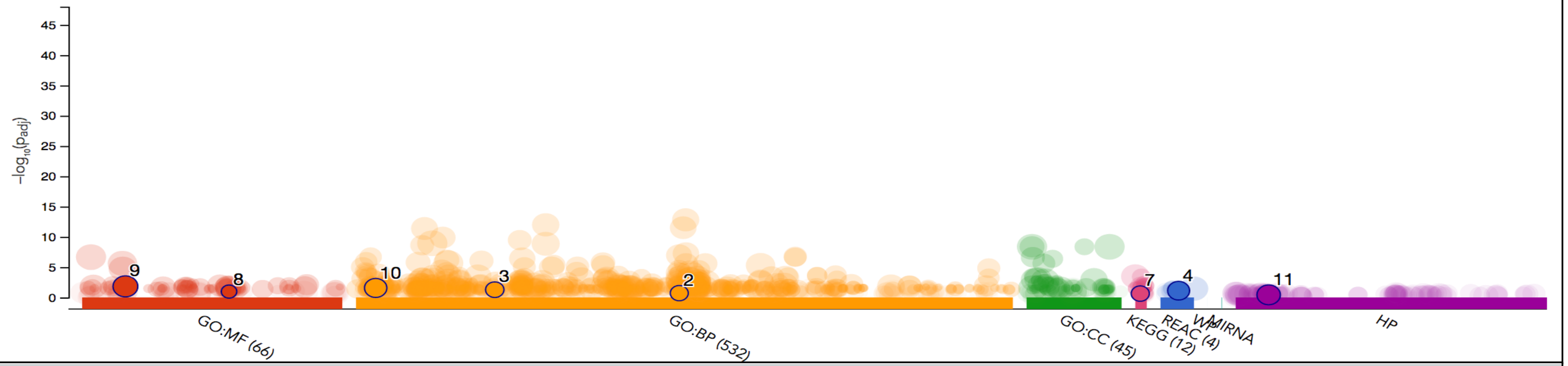

ID	Source	Term ID	Term Name	p_{adj} (5_DPI_PRRSV_UP)	p_{adj} (5_DPI_PRRSV_DOWN)
1	GO:BP	GO:0002699	positive regulation of immune effector process	2.941×10^{-5}	
2	GO:BP	GO:0048525	negative regulation of viral process	1.560×10^{-7}	~~1.972×10^{-1}~~
3	GO:BP	GO:0019079	viral genome replication	9.294×10^{-5}	~~5.331×10^{-2}~~
4	REAC	REAC:R-SSC-6798695	Neutrophil degranulation	2.239×10^{-4}	~~7.712×10^{-2}~~
5	GO:BP	GO:0030593	neutrophil chemotaxis	3.386×10^{-5}	
6	GO:BP	GO:0032481	positive regulation of type I interferon production	5.674×10^{-5}	
7	KEGG	KEGG:04061	Viral protein interaction with cytokine and cytokine receptor	6.961×10^{-9}	~~2.333×10^{-1}~~
8	GO:MF	GO:0043394	proteoglycan binding	4.236×10^{-6}	~~1.081×10^{-1}~~
9	GO:MF	GO:0008144	drug binding	3.521×10^{-2}	1.498×10^{-2}
10	GO:BP	GO:0002252	immune effector process	2.227×10^{-16}	2.742×10^{-2}
11	HP	HP:0002088	Abnormal lung morphology	4.014×10^{-7}	~~3.740×10^{-1}~~
12	HP	HP:0002205	Recurrent respiratory infections	7.736×10^{-6}	

PRRSV VS. Control 7 DPI: G.O. Multi-query

> 7DPI_PRRSV_UP

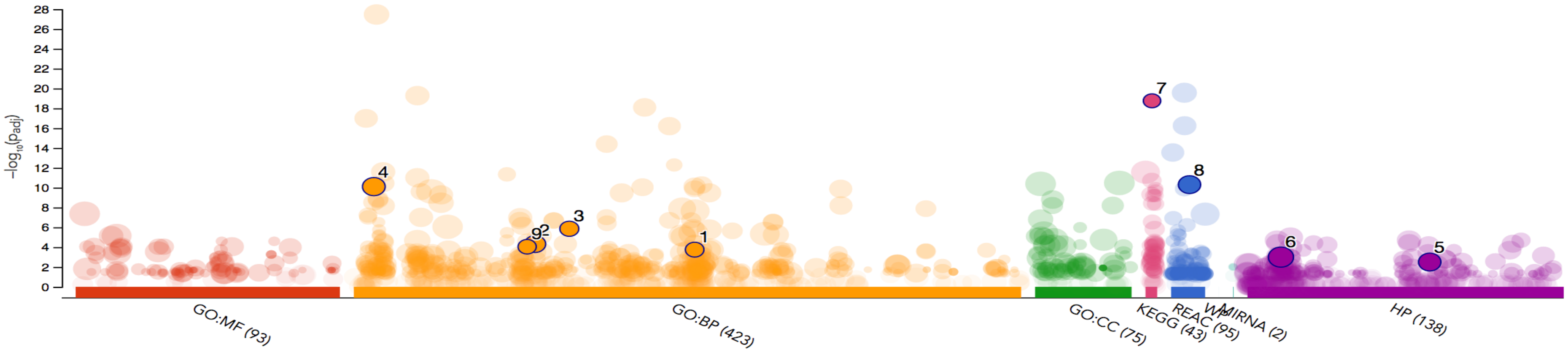

> 7_DPI_PRRSV_DOWN

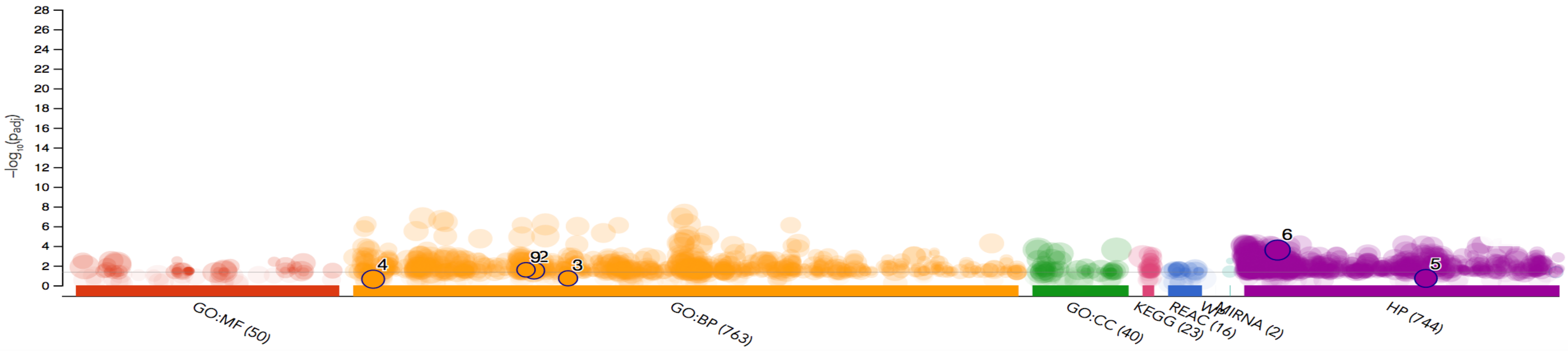

ID	Source	Term ID	Term Name	p_{adj} (7DPI_PRRSV_UP)	p_{adj} (7_DPI_PRRSV_DOWN)
1	GO:BP	GO:0050868	negative regulation of T cell activation	1.795×10^{-4}	
2	GO:BP	GO:0031349	positive regulation of defense response	4.869×10^{-5}	3.175×10^{-2}
3	GO:BP	GO:0034341	response to interferon-gamma	1.399×10^{-6}	~~1.931×10^{-1}~~
4	GO:BP	GO:0002252	immune effector process	7.933×10^{-11}	~~2.374×10^{-1}~~
5	HP	HP:0011947	Respiratory tract infection	3.189×10^{-3}	~~1.980×10^{-1}~~
6	HP	HP:0002086	Abnormality of the respiratory system	1.011×10^{-3}	2.623×10^{-4}
7	KEGG	KEGG:04612	Antigen processing and presentation	1.754×10^{-19}	
8	REAC	REAC:R-SSC-6798695	Neutrophil degranulation	4.983×10^{-11}	
9	GO:BP	GO:0030593	neutrophil chemotaxis	9.103×10^{-5}	2.646×10^{-2}

IBV/PRRSV VS. Control 3 DPI: G.O. Multi-query

> 3DPI_FLUB/PRRSV_UP

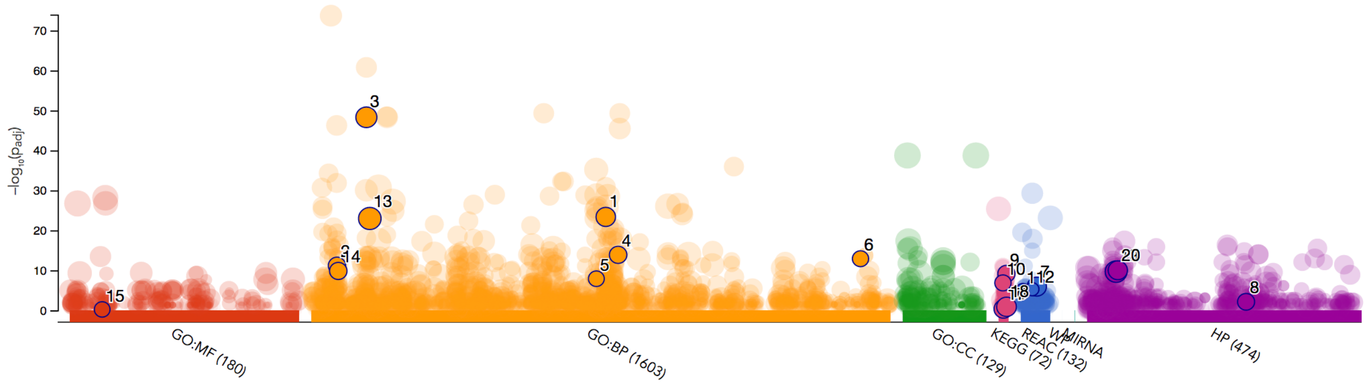

> 3DPI_FLUB/PRRSV_DOWN

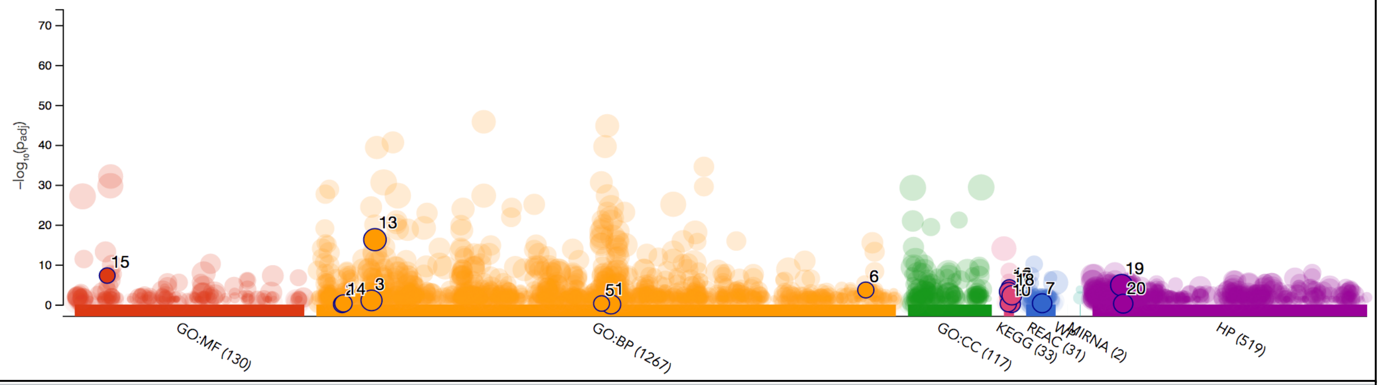

ID	Source	Term ID	Term Name	p_{adj} (3DPI_FLUB/PRRSV_UP)	p_{adj} (3DPI_FLUB/PRRSV_DOWN)
1	GO:BP	GO:0050778	positive regulation of immune response	4.313×10^{-24}	~~9.298×10^{-1}~~
2	GO:BP	GO:0002699	positive regulation of immune effector process	6.705×10^{-12}	~~7.916×10^{-1}~~
3	GO:BP	GO:0006952	defense response	5.123×10^{-49}	~~9.349×10^{-2}~~
4	GO:BP	GO:0051607	defense response to virus	1.324×10^{-14}	
5	GO:BP	GO:0048525	negative regulation of viral process	1.257×10^{-8}	~~5.503×10^{-1}~~
6	GO:BP	GO:1990266	neutrophil migration	1.240×10^{-13}	2.243×10^{-4}
7	REAC	REAC:R-SSC-6798695	Neutrophil degranulation	1.178×10^{-6}	~~4.692×10^{-1}~~
8	HP	HP:0011991	Abnormal neutrophil count	7.492×10^{-3}	
9	KEGG	KEGG:05164	Influenza A	7.111×10^{-10}	~~7.645×10^{-1}~~
10	KEGG	KEGG:04064	NF-kappa B signaling pathway	1.391×10^{-7}	~~6.466×10^{-1}~~
11	REAC	REAC:R-SSC-1169410	Antiviral mechanism by IFN-stimulated genes	9.203×10^{-6}	
12	REAC	REAC:R-SSC-1169408	ISG15 antiviral mechanism	5.248×10^{-6}	
13	GO:BP	GO:0007166	cell surface receptor signaling pathway	1.059×10^{-23}	5.600×10^{-17}
14	GO:BP	GO:0002768	immune response-regulating cell surface receptor signaling pathway	1.423×10^{-10}	~~5.073×10^{-1}~~
15	GO:MF	GO:0005201	extracellular matrix structural constituent	~~5.960×10^{-1}~~	5.576×10^{-8}
16	KEGG	KEGG:04512	ECM-receptor interaction	~~1.589×10^{-1}~~	5.622×10^{-5}
17	KEGG	KEGG:04151	PI3K-Akt signaling pathway	~~4.536×10^{-1}~~	5.930×10^{-4}
18	KEGG	KEGG:05200	Pathways in cancer	~~1.332×10^{-1}~~	4.758×10^{-3}
19	HP	HP:0002086	Abnormality of the respiratory system	2.065×10^{-10}	1.372×10^{-5}
20	HP	HP:0002205	Recurrent respiratory infections	9.635×10^{-11}	~~6.057×10^{-1}~~

IBV/PRRSV VS. Control 5 DPI: G.O. Multi-query

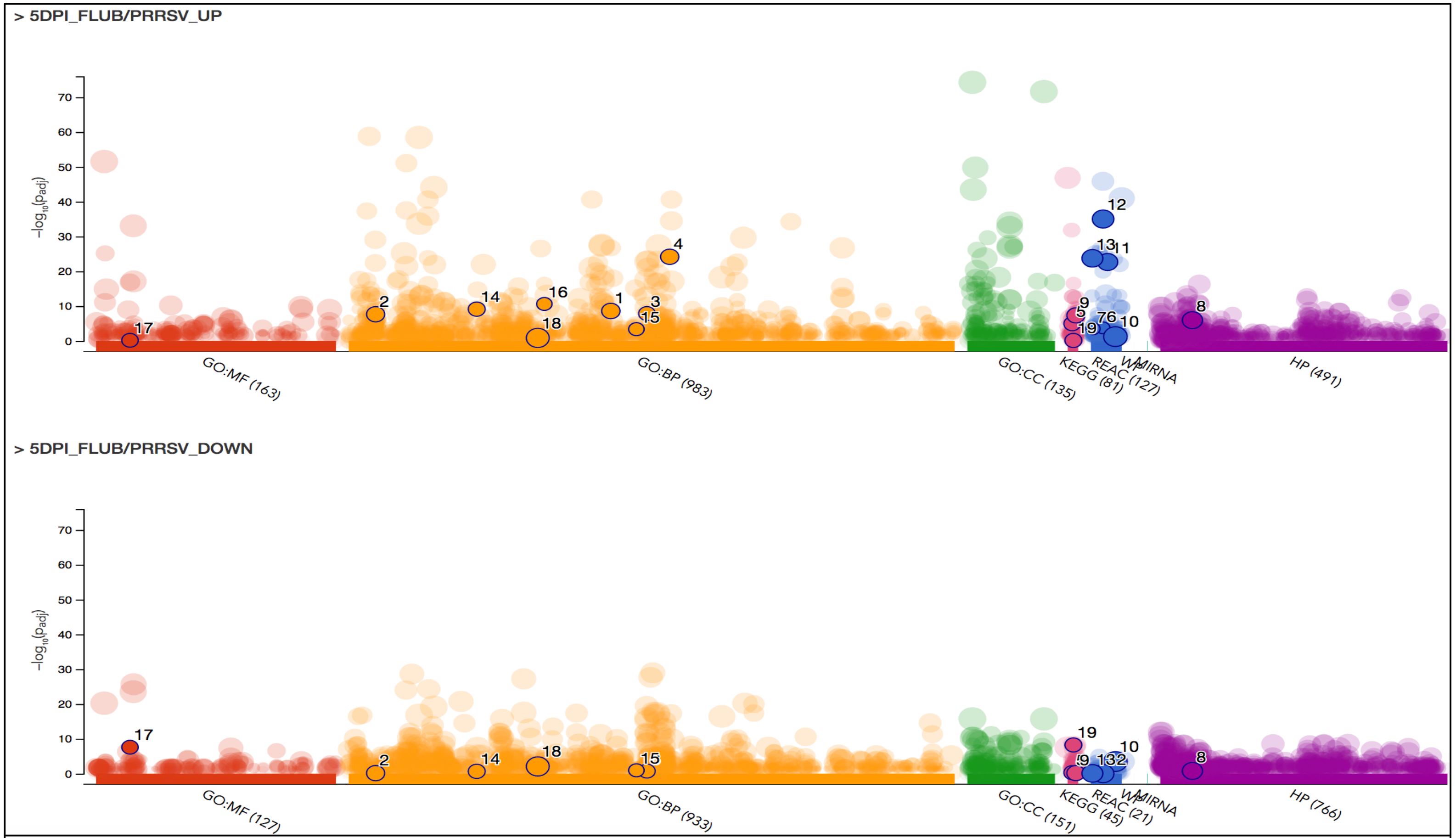

ID	Source	Term ID	Term Name	p_{adj} (5DPI_FLUB/PRRSV_UP)	p_{adj} (5DPI_FLUB/PRRSV_DOWN)
1	GO:BP	GO:0045089	positive regulation of innate immune response	2.571×10^{-9}	
2	GO:BP	GO:0002699	positive regulation of immune effector process	2.221×10^{-8}	~~7.233×10^{-1}~~
3	GO:BP	GO:0048525	negative regulation of viral process	1.394×10^{-8}	~~1.858×10^{-1}~~
4	GO:BP	GO:0051607	defense response to virus	6.829×10^{-25}	
5	KEGG	KEGG:04061	Viral protein interaction with cytokine and cytokine receptor	1.194×10^{-5}	~~5.230×10^{-1}~~
6	REAC	REAC:R-SSC-1169408	ISG15 antiviral mechanism	1.736×10^{-4}	
7	REAC	REAC:R-SSC-1169410	Antiviral mechanism by IFN-stimulated genes	3.162×10^{-4}	
8	HP	HP:0002205	Recurrent respiratory infections	1.108×10^{-6}	~~1.696×10^{-1}~~
9	KEGG	KEGG:05164	Influenza A	4.685×10^{-8}	~~7.042×10^{-1}~~
10	REAC	REAC:R-SSC-162582	Signal Transduction	~~5.938×10^{-2}~~	3.329×10^{-4}
11	REAC	REAC:R-SSC-6798695	Neutrophil degranulation	2.189×10^{-23}	
12	REAC	REAC:R-SSC-168249	Innate Immune System	9.776×10^{-36}	~~9.982×10^{-1}~~
13	REAC	REAC:R-SSC-1280218	Adaptive Immune System	1.899×10^{-24}	~~8.520×10^{-1}~~
14	GO:BP	GO:0019079	viral genome replication	7.604×10^{-10}	~~2.227×10^{-1}~~
15	GO:BP	GO:0046718	viral entry into host cell	4.257×10^{-4}	~~1.117×10^{-1}~~
16	GO:BP	GO:0034340	response to type I interferon	2.430×10^{-11}	
17	GO:MF	GO:0005201	extracellular matrix structural constituent	~~6.711×10^{-1}~~	2.570×10^{-8}
18	GO:BP	GO:0033554	cellular response to stress	~~1.376×10^{-1}~~	8.551×10^{-3}
19	KEGG	KEGG:04512	ECM-receptor interaction	~~7.465×10^{-1}~~	5.813×10^{-9}

IBV/PRRSV VS. Control 7 DPI: G.O. Multi-query

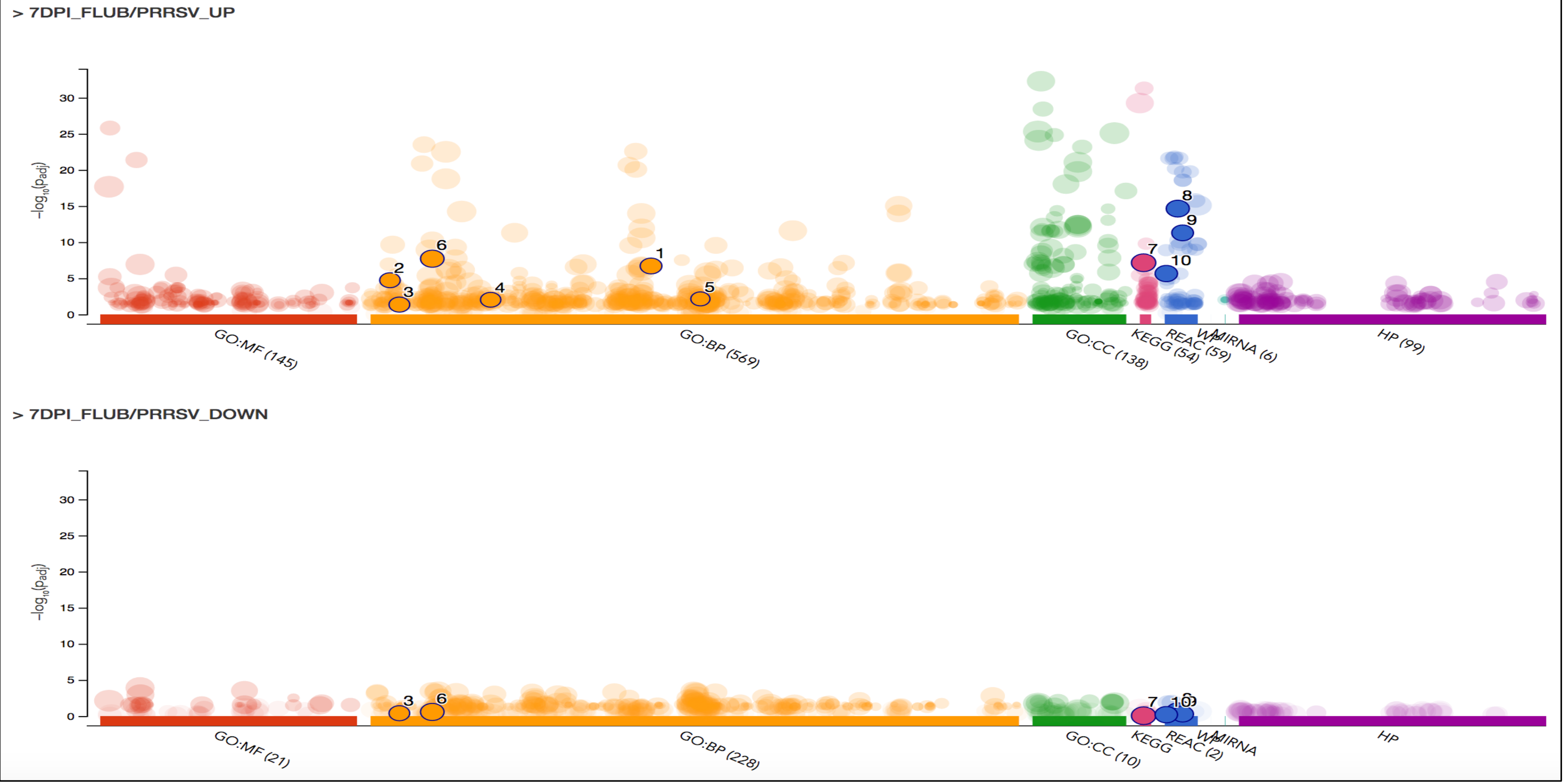

ID	Source	Term ID	Term Name	p_{adj} (7DPI_FLUB/PRRSV_UP)	p_{adj} (7DPI_FLUB/PRRSV_DOWN)
1	GO:BP	GO:0045087	innate immune response	2.009×10^{-7}	
2	GO:BP	GO:0002250	adaptive immune response	1.887×10^{-5}	
3	GO:BP	GO:0002697	regulation of immune effector process	4.455×10^{-2}	~~4.050×10^{-1}~~
4	GO:BP	GO:0016032	viral process	9.551×10^{-3}	
5	GO:BP	GO:0050792	regulation of viral process	7.223×10^{-3}	
6	GO:BP	GO:0006952	defense response	1.976×10^{-8}	~~2.695×10^{-1}~~
7	KEGG	KEGG:01100	Metabolic pathways	7.135×10^{-8}	~~8.986×10^{-1}~~
8	REAC	REAC:R-SSC-168249	Innate Immune System	2.281×10^{-15}	~~2.168×10^{-1}~~
9	REAC	REAC:R-SSC-6798695	Neutrophil degranulation	5.226×10^{-12}	~~5.349×10^{-1}~~
10	REAC	REAC:R-SSC-1280218	Adaptive Immune System	2.235×10^{-6}	~~6.454×10^{-1}~~
